# Activation of the Anrep Effect in Aortic Stenosis Pre-TAVR and Post-TAVR: An Echocardiographic Pressure-Volume Analysis

**DOI:** 10.1016/j.jacadv.2025.102424

**Published:** 2025-12-17

**Authors:** Jan-Christian Reil, Vasco Sequeira, Philipp Lucas, Lea Tadros, Gert-Hinrich Reil, Jan M. Federspiel, Smita Scholtz, Hazem Omran, Paul Steendijk, Christoph Marquetand, Werner Scholtz, Cornelia Piper, Tanja Rudolph, Volker Rudolph

**Affiliations:** a Klinik für allgemeine und interventionelle Kardiologie, Herz-und Diabetes Zentrum Nordrhein-Westphalen, Bad Oeynhausen, Germany; b Comprehensive Heart Failure Center, University Clinic Würzburg, Würzburg, Germany; c Universitätsklinik für Innere Medizin – Kardiologie, Klinikum Oldenburg, Oldenburg, Germany; d Institute for Legal Medicine, Faculty of Medicine, Saarland University, Homburg, Saarland, Germany; e Department of Cardiology, Leiden University Medical Center, Leiden, the Netherlands; f Department of Cardiology, Medical Clinic II, University Heart Center Lübeck, Lübeck, Germany

**Keywords:** afterload reduction, Anrep effect, aortic stenosis, LV contractility, pressure-volume analysis, transcatheter aortic valve replacement (TAVR)

## Abstract

**Background:**

In aortic stenosis (AS), chronic pressure overload of the left ventricle (LV) may sustain an intrinsic, afterload-dependent adaptive response known as the Anrep effect, characterized by increased myocardial contractility and prolonged systolic duration. Whether this response resolves following transcatheter aortic valve replacement (TAVR) remains unknown.

**Objectives:**

The objective of the study was to determine whether the Anrep is chronically activated in severe AS and acutely reverses after TAVR.

**Methods:**

We studied 119 patients with high-gradient AS undergoing TAVR. Pressure-volume (PV) loops were analyzed by echocardiography before and 24-hours after intervention. The “Anrep triad”, defined as elevated afterload (LV end-systolic pressure, effective arterial elastance), enhanced contractility (end-systolic elastance, end-systolic volume at 150 mm Hg), and prolonged systolic ejection time, was assessed. Stroke work (SW), potential energy, PV area (PVA), and mechanical efficiency (SW/PVA) quantified LV energetics.

**Results:**

TAVR reduced afterload (LV end-systolic pressure: 220 vs 143 mm Hg; effective arterial elastance: 2.8 vs 2.0 mm Hg/mL) and contractility (end-systolic elastance: 4.5 vs 2.6 mm Hg/mL; end-systolic volume at 150 mm Hg: 39 vs 53 mL), while shortening systolic duration (systolic ejection time: 390 vs 321 ms) (all *P* < 0.0001). LV ejection fraction was unchanged (56% vs 56%, *P* = 0.44). Mechanical workload decreased (SW: 9,017 vs 6,257 mm Hg mL; PVA: 14,261 vs 10,017 mm Hg mL, *P* < 0.0001), while efficiency was preserved (64% vs 63%, *P* = 0.101).

**Conclusions:**

In severe AS, the Anrep effect supports output at high energetic cost. TAVR reverses this state, unloading the heart and reducing mechanical demand without altering ejection fraction. This identifies the Anrep as a clinically relevant load-dependent mechanism in AS and highlights its reversibility post-TAVR.

Aortic stenosis (AS) is the most common valvular heart disease in aging populations, primarily caused by calcific degeneration of trileaflet or congenital bicuspid valves.^1^^,^^2^ Progressive narrowing of the valve increases systolic pressure load on the left ventricle (LV), triggering compensatory hypertrophy to normalize wall stress.^3^^,^^4^ Initially, this geometric adaptation is accompanied by enhanced systolic performance, supported by intrinsic inotropic mechanisms that respond to increased afterload. Over time, however, sustained afterload leads to maladaptive remodeling, including myocardial fibrosis, diastolic dysfunction, and energetic inefficiency, even in patients with preserved ejection fraction (EF), ultimately increasing the risk of heart failure.^5^

Transcatheter aortic valve replacement (TAVR), introduced in 2002 by Cribier et al,^6^ has transformed the management of symptomatic AS.^7^ Once limited to high-risk or inoperable patients,^8^ TAVR is now widely used across risk categories and provides rapid hemodynamic relief.^9^^,^^10^ Although it effectively reduces transvalvular gradients and alleviates symptoms, its impact on intrinsic myocardial adaptations to chronic pressure overload, particularly contractile and energetic responses, remains incompletely characterized.

One such key compensatory mechanism is the Anrep effect, a physiological response in which acute afterload elevation increases contractility and prolongs systolic ejection to maintain or increase stroke volume.^11^^,^^12^ We recently showed that this mechanism persists chronically in pressure-overloaded states, evolving into an energetically costly strategy to preserve output.^13^^,^^14^ For instance, in obstructive hypertrophic cardiomyopathy (oHCM), a condition marked by left ventricular (LV) outflow tract obstruction and hyperdynamic contraction (EF >70%), we identified a chronic “Anrep phenotype” characterized by elevated contractility, prolonged systolic duration, and increased energy demand.^14^ All these features reversed after targeted afterload reduction via alcohol septal ablation.^13^^,^^14^ AS presents a parallel scenario, wherein prolonged LV pressure overload may chronically activate the Anrep effect. Whether TAVR reverses this adaptation remains unknown.

To address this, we conducted noninvasive echocardiographic pressure-volume (PV) loop analysis in 119 patients with severe AS undergoing transfemoral TAVR. We assessed Anrep-related parameters, the so-called “Anrep triad”, before and after intervention. This triad includes: 1) elevated afterload indices, namely LV end-systolic pressure (LVESP), and effective arterial elastance (Ea); 2) increased contractility markers, reflected by a steeper end-systolic PV relationship (ESPVR) and its slope end-systolic elastance (Ees); and 3) prolonged systolic duration, measured as systolic ejection time (SET).^13^^,^^14^ We additionally assessed myocardial workload and oxygen demand using stroke work (SW), PV area (PVA), and mechanical efficiency (SW/PVA).^14^ By integrating these parameters, we tested the hypothesis that the Anrep effect is chronically activated in AS and that TAVR acutely reverses this adaptation, offering a novel mechanism for myocardial recovery following afterload reduction.

## Methods

### Patient population

We studied 119 patients with severe, symptomatic AS who were scheduled to undergo TAVR at our center. The screening and enrollment flow is shown in Supplemental Figure 1. Severe AS was defined as an aortic valve opening area <1.0 cm^2^ on echocardiography, in accordance with current guidelines.^15^ Patients had either normal-flow high-gradient AS (stroke volume index ≥35 mL/m^2^, mean gradient ≥40 mm Hg) or paradoxical low-flow low-gradient AS (stroke volume index <35 mL/m^2^, mean gradient <40 mm Hg) with preserved EF (EF ≥50%). The cohort consisted predominantly of patients with higher age, with 93% aged 75 years or older and only 7% between 65 and 74 years. Exclusion criteria included LV EF (LVEF) <50% (to avoid confounding contractile impairment unrelated to afterload compensation), more than mild aortic regurgitation, significant concomitant valvular disease, uncontrolled hypertension, unsuccessful valve deployment, persistent significant postprocedural gradients, or acute complications such as annular rupture or device embolization. This was a single-center, retrospective analysis; however, all patients provided prospective written informed consent for the use of their clinical and echocardiographic data for research purposes. Patients were enrolled between January and May 2024 at the Heart and Diabetes Center North Rhine-Westphalia, Bad Oeynhausen, Germany. The study was approved by the internal review board of the Herz-und Diabeteszentrum Nordrhein-Westfalen, Bad Oeynhausen, Germany, and all patients provided written informed consent before participation.

Severe AS was diagnosed using comprehensive transthoracic echocardiography. Baseline and postprocedure assessments were performed 1 day before and 1 day after TAVR, respectively. The aortic valve opening area was calculated using the continuity equation, in which the LV outflow tract diameter was measured in the parasternal long-axis view at mid-systole to derive the cross-sectional area. The LV outflow tract velocity-time integral (VTI) was obtained using pulsed-wave Doppler just beneath the aortic valve, and the transvalvular aortic valve VTI was measured in the five-chamber view using continuous-wave Doppler, paying attention to a small angular error. The aortic valve area was then calculated as the product of the LV outflow tract cross-sectional area and the LV outflow tract VTI, divided by the transvalvular aortic valve VTI.

### TAVR procedure

All patients underwent TAVR using standard transfemoral techniques with contemporary prosthetic valves. The choice of transcatheter valve type, including balloon-expandable Sapien (Edwards), self-expanding Evolut (Medtronic), or Accurate (Boston Scientific), was determined by the heart team based on anatomical considerations. Postimplantation, mean aortic valve gradients were reduced to <20 mm Hg in all patients, confirming effective afterload relief.

### Echocardiographic pressure-volume loop analysis

**Figure 1:**
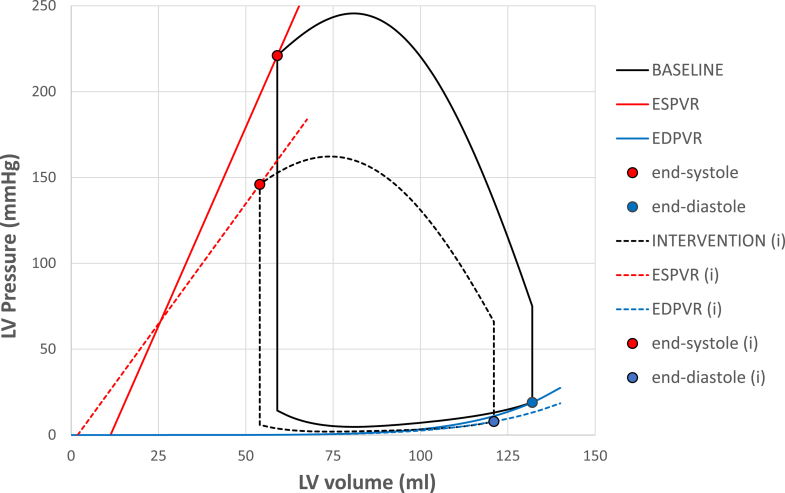
**Group-Averaged Pressure-Volume Loops Before and After TAVR** Pressure-volume (PV) loops represent group-averaged data from the full study cohort, reconstructed using echocardiography-based methods before (solid black loop) and after transcatheter aortic valve replacement (TAVR) (dashed black loop). End-systolic PV relationship (ESPVR) (red lines) and end-diastolic PV relationship (EDPVR) (blue lines) are shown for both conditions. Solid and dashed symbols represent end-systolic and end-diastolic points, respectively, before (●, ◯) and after intervention (●(i), ◯(i)). Post-TAVR loops demonstrate a rightward and downward shift in ESPVR and shortening of systolic duration, consistent with reversal of the Anrep effect. LV = left ventricular.

**Figure 2:**
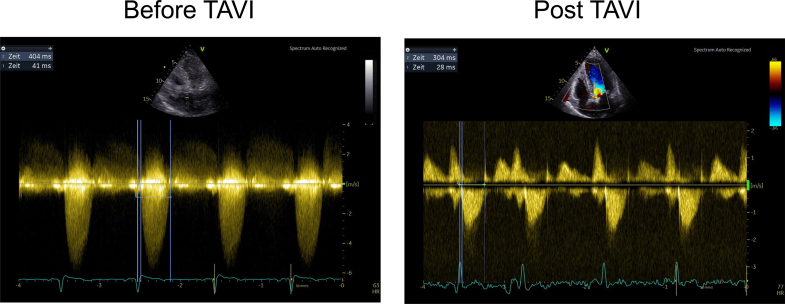
**Normalization of Systolic Ejection Time Following TAVR** Continuous-wave Doppler recordings of transaortic flow from an apical 5-chamber view before (left panel) and after (right panel) transcatheter aortic valve replacement (TAVR). Systolic ejection time (SET) was measured as the interval between the electrocardiography R-wave and cessation of forward aortic flow (blue vertical lines). An ∼100 ms shortening of SET is evident post-TAVR, consistent with reduced contractile demand and resolution of prolonged systole previously required to overcome fixed afterload.

Hemodynamic assessments were performed 1 day before and within 72 hours after TAVR. LV function was analyzed using echocardiography-based PV loop assessment. Figure 1 presents the group-averaged PV loop and the key measurements derived. Contractility was estimated using the ESPVR, calculated via the established single-beat method according to the approach by Chen et al.^16^ This relationship was defined by its slope, Ees, and its intercept with the volume axis (V_0_).^17^ The end-systolic volume at a fixed pressure of 150 mm Hg (ESV_150_) was calculated using these parameters. Diastolic function was assessed using the single-beat end-diastolic PV relationship, based on the validated method from Klotz et al.^18^ This curve was described by the equation P_ed_ = *α* × EDV^β^, where P_ed_ is the LV end-diastolic pressure (EDP) (LVEDP), *α* is a curve-fitting constant, *β* reflects ventricular stiffness, and EDV is the end-diastolic volume. LVEDP was estimated from the mitral inflow velocity (E) and early diastolic mitral annular tissue velocity (E') using the formula: LVEDP = 11.96 + 0.596 × E/E'.^19^ This provides a noninvasive estimate of LV filling pressure. To estimate ventricular capacitance, we determined the EDV corresponding to an LVEDP of 15 mm Hg (termed V_15_). A rightward shift in the end-diastolic PV relationship increases V_15_, whereas a leftward shift decreases it.^18^ Resting brachial blood pressure was measured using a standard arm cuff. Afterload was quantified as the LVESP, calculated as the sum of systolic cuff pressure and the peak aortic valve Doppler gradient. Ea was then derived by dividing LVESP by stroke volume. Systolic performance was assessed using Ees, ESV_150_, and SET. SET was measured from apical 5-chamber Doppler recordings as the interval between the electrocardiography R-wave and the end of the aortic outflow Doppler signal (Figure 2). Heart rate was determined from the R-R interval and used to adjust SET using the Fridericia formula: heart rate–adjusted SET (SET_c_) = SET/(R-R^1/3^), allowing comparisons at a normalized rate of 60 bpm.^20^ To assess LV work and energy use, we calculated SW, potential energy (PE), and the total PVA. SW corresponds to the area within the PV loop, whereas PE refers to the triangular area between the ESPVR, the volume axis, and the PV loop. PVA was calculated as the sum of SW and PE (PVA = SW + PE) and served as a surrogate for myocardial oxygen demand.^21^ LV mechanical efficiency was defined as the ratio of SW to PVA (SW/PVA).

### Statistics

Pre/postanalyses used paired observations. Paired data were available in 119 for all prespecified endpoints except diastolic indices requiring tissue-Doppler E′ (LVEDP from E/E′) and derived EDV at 15 mm Hg, which were available as paired data in 93 because E′ was unavailable or suboptimal at 1 of the 2 time points. Continuous variables were reported as medians with IQRs (Q1–Q3). Distribution normality was assessed using the Shapiro-Wilk test. Hemodynamic and echocardiographic parameters before and after TAVR were compared using the Wilcoxon signed-rank test for paired data. A *P* value <0.05 was considered statistically significant. All analyses were conducted using GraphPad Prism version 7.05 (GraphPad Software).

## Results

### Patient characteristics

In this study, 119 patients with severe AS underwent TAVR to relieve the afterload gradient. Baseline clinical characteristics are summarized in Table 1, and preprocedure and postprocedure hemodynamic parameters are presented in Table 2. The cohort had a median peak gradient across the LV outflow tract and aortic valve of 74 (65-86) mm Hg and a baseline aortic valve area of 0.7 (0.5-0.9) cm^2^. LVEF was preserved (56% [52% to 60%]) despite chronic pressure overload, consistent with the activation of compensatory mechanisms. Additional baseline parameters included a heart rate of 66 (60-73) beats/min and a stroke volume of 73 (62-82) mL (Table 2). Most patients were symptomatic, classified as NYHA functional class II or III. No periprocedural myocardial infarctions or hemodynamically significant arrhythmias occurred. Post-TAVR echocardiography confirmed correctly positioned prosthetic valves with either trivial or no paravalvular regurgitation.

**Table 1:** Baseline Clinical Characteristics of Patients With Aortic Stenosis Undergoing TAVR (N = 119)

Demographics	
Age ≥75 y	111 (93%)
Age 65-74 y	8 (7%)
Median age	75 [65-84]
Male	62 (52%)
Female	58 (48%)
Comorbidities	
Coronary artery disease	59
Hypertension	99
Diabetes	22
Dyslipidemia	96
Smoking history	25
Body mass index	
BMI (kg/m^2^)	26.7 [24.7-29.7]
Medications	
Beta-blockers	62
ACE inhibitors	31
ARBs	43
ARNi	3
Diuretics	69
Aldosterone antagonists	14
SGLT2 inhibitors	19
Valve type	
Sapien (Edwards)	36 (30%)
Accurate (Boston Scientific)	24 (20%)
Evolut (Medtronic)	59 (50%)
NYHA functional class	
I	17 (14%)
II	53 (45%)
III	49 (41%)

ACE = angiotensin-converting enzyme; ARBs = angiotensin II receptor blockers; ARNi = angiotensin receptor-neprilysin inhibitors; BMI = body mass index; SGLT2 = sodium-glucose cotransporter 2; TAVR = transcatheter aortic valve replacement.

**Table 2:** Changes in Key Clinical Parameters in AS Patients Pre-TAVR and Post-TAVR (N = 119)

Parameter	Pre-TAVR	Post-TAVR	*P* Value
General			
Heart rate (beats/min)	66 [60-73]	72 [65-80]	<0.0001
Ejection fraction (%)	56 [52-60]	56 [54-60]	0.44
Stroke volume (mL)	73 [62-82]	67 [56-79]	<0.0001
Afterload			
AV gradient (mm Hg)	74 [65-86]	14 [10-20]	<0.0001
ESP (mm Hg)	220 [197-244]	143 [132-160]	<0.0001
Ea (mm Hg/mL)	2.8 [2.4-3.3]	2.0 [1.7-2.43]	<0.0001
Contractility			
Ees (mm Hg/mL)	4.5 [3.5-5.7]	2.6 [1.9-3.5]	<0.0001
ESV_150_ (mL)	39 [31-52]	53 [43-65]	<0.0001
Systolic duration			
SET (ms)	390 [362-407]	321 [297-355]	<0.0001
SET_c_ (ms)	396 [374-419]	346 [319-372]	<0.0001
Energy and efficiency			
SW (mm Hg·mL)	9,017 [7,545-10,328]	6,257 [5,077-7,705]	<0.0001
PE (mm Hg·mL)	4,955 [4,019-6,191]	3,639 [2,774-4,750]	<0.0001
PVA (mm Hg·mL)	14,261 [11,754-16,609]	10,017 [8,069-12,608]	<0.0001
ME (SW/PVA) (%)	64 [61-68]	63 [58-69]	0.101
Diastolic function			
EDP (mm Hg) (N = 93)	18.9 [13.9-23.9]	14.8 [9.5-21.9]	<0.0001
EDV (mL)	132 [112-150]	120 [103-141]	<0.0001
EDV_15_ (mL) (N = 93)	122 [106-147]	124 [96-150]	0.51

AS = aortic stenosis; AV Gradient = transvalvular aortic valve gradient; Ea = effective arterial elastance; EDP = end-diastolic pressure; EDV = end-diastolic volume; EDV_15_ = end-diastolic volume at 15 mm Hg. Pre/post comparisons use paired data; Ees = end-systolic elastance; ESP = left ventricular end-systolic pressure; ESV_150_ = end-systolic volume at an estimated pressure of 150 mm Hg; ME = mechanical efficiency; PE = potential energy; PVA = pressure-volume area; SET = systolic ejection time; SET_c_ = heart rate–corrected systolic ejection time; SW = stroke work; TAVR = transcatheter aortic valve replacement.

Values are median [Q1-Q3]. All variables have 119 paired observations except EDP and EDV_15_, which have 93 due to unavailable or suboptimal tissue-Doppler E′ at 1 of the 2 time points.

### Preprocedural and postprocedural valvular gradient and afterload

TAVR led to an immediate and marked reduction in the aortic valve gradient: the peak transaortic gradient decreased from 74 (65-86) to 14 (10-20) mm Hg (*P* < 0.0001), indicating effective relief of valvular obstruction (Table 2). This was accompanied by a parallel decline in afterload: LVESP decreased from 220 (197-244) to 143 (132-160) mm Hg (*P* < 0.0001), and Ea from 2.8 (2.4-3.3) to 2.0 (1.7-2.4) mm Hg/mL (*P* < 0.0001). The substantial drop in peak systolic pressure reflects significant LV unloading, whereas the reduction in Ea, which accounts for both valvular and arterial components, suggests improved ventriculoarterial coupling primarily due to relief of valve obstruction. Notably, heart rate increased post-TAVR (66 [60-73] to 72 [65-80] beats/min, *P* < 0.0001), likely reflecting relief of baroreflex inhibition or mild procedural stress. However, this change did not influence the assessment of afterload, as PV parameters were adjusted for heart rate where appropriate.

### Contractility and systolic function

**Figure 3:**
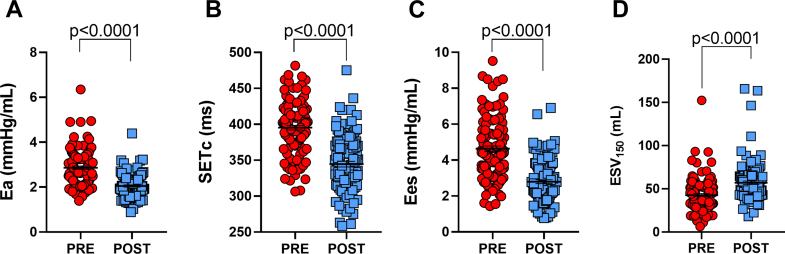
**Quantitative Reversal of the Anrep Triad After Transcatheter Aortic Valve Replacement** Bar graphs show group-level changes in the 3 hallmark indices of the Anrep effect: (1) effective arterial elastance (Ea), a marker of afterload; (2) heart rate–corrected systolic ejection time (SET_c_); and (3) 2 indicators of contractility, end-systolic elastance (Ees) and end-systolic volume at 150 mm Hg (ESV_150_). Red bars indicate pre-TAVR values; blue bars represent post-TAVR values. All indices show significant reversal postintervention (*P* < 0.0001), reflecting the acute deactivation of the load-dependent contractile state.

Indices of LV contractility showed significant changes consistent with unloading of the previously enhanced inotropic state. After TAVR, the ESPVR shifted downward and rightward (Figure 1), Ees decreased from 4.5 (3.5-5.7) to 2.6 (1.9-3.5) mm Hg/mL (*P* < 0.0001), and ESV_150_ increased from 39 (31-52) to 53 (43-65) mL (*P* < 0.0001) (Table 2, Figure 3). These findings indicate that the LV generated less force for a given volume postTAVR. LVEF remained essentially unchanged (56% [52% to 60%] vs 56% [54% to 60%], *P* = 0.44), and stroke volume modestly declined (73 [62-82] to 67 [56-79] mL, *P* < 0.0001) (Table 2). These results are consistent with prior reports showing that TAVR does not acutely raise EF.^22^

### Systolic ejection duration

Concomitant with changes in contractility, systolic duration was significantly reduced post-TAVR. LV ejection time (SET) decreased from 390 (362-407) ms to 321 (297-355) ms (*P* < 0.0001), and SET_c_ shortened from 396 (374-419) to 346 (319-372) ms (*P* < 0.0001) (Table 2, Figure 3). This reflects faster and more efficient ejection through the now-unobstructed aortic valve and a reduced requirement for prolonged contraction. The combined shortening of SET and decline in Ees indicate that LV contraction became both briefer and less forceful once afterload was removed.

**Figure 4:**
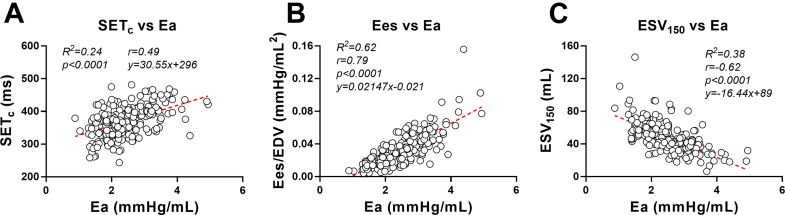
**Correlations Between Afterload and Contractility Markers in Aortic Stenosis** Scatter plots show linear regressions between effective arterial elastance (Ea) (x-axis) and 3 contractile indices (y-axis): SET_c_, Ees/EDV, and ESV_150_. Red dashed lines represent regression fits. Strong positive correlations between Ea and Ees, and inverse correlations with ESV_150_, indicate that contractility scales with afterload in AS. These relationships support the presence of a load-dependent inotropic adaptation consistent with the chronic Anrep effect. AS = aortic stenosis; SETc = heart rate–corrected systolic ejection time; Ees = end-systolic elastance; EDV = end-diastolic volume; ESV_150_ = end-systolic volume at 150 mm Hg.

These findings confirm that the Anrep mechanism, typically transient in healthy physiology, becomes chronically activated in severe AS due to sustained afterload (Ea). This is reflected in persistently elevated Anrep triad indices (afterload, contractility, systolic duration) before TAVR and their acute reversal postprocedure (Figures 3A to 3D). Further support is provided by the data presented in Figure 4, where SET_c_, Ees, and ESV_150_ show the strongest correlations with Ea (Figures 4A to 4C) underscoring the tight coupling between afterload and these contractile markers. Additional associations with energy-related indices, including SW, PVA, and PE, are shown in Supplemental Figures 2A to 2C, highlighting a broader link between afterload and myocardial energetic demand.

### Mechanical energy and work

Although the Anrep response is compensatory, it is energetically costly. Pre-TAVR, patients had elevated mechanical workload, which significantly decreased following reversal of the Anrep effect. After TAVR, SW, the energy the LV expends to eject blood each beat, declined from 9,017 (7,545-10,328) to 6,257 (5,077-7,705) mm Hg·mL (*P* < 0.0001) (Table 2). This large reduction is expected given the drop in LVESP (a key determinant of SW) and the slight reduction in stroke volume. PE, the residual stored energy at end-systole, decreased from 4,955 (4,019-6,191) to 3,639 (2,774-4,750) mm Hg·mL (*P* < 0.0001) (Table 2). Total PVA (PVA = SW + PE), a surrogate for myocardial oxygen demand,^21^ was significantly lower post-TAVR (10,017 [8,069-12,608] vs 14,261 [11,754-16,609] mm Hg·mL, *P* < 0.0001) (Table 2). These results indicate that LV oxygen demand and mechanical stress were acutely ameliorated by unloading. Despite these reductions, mechanical efficiency (SW/PVA) did not change (64% [61%-68%] pre vs 63% [58%-69%] post, *P* = 0.101), indicating that energy used for forward output remained proportionate to total expenditure. In essence, the heart performed less work without compromising efficiency.

### Diastolic function

Early improvements in diastolic filling were also observed. LVEDP decreased from 18.9 (13.9-23.9) to 14.8 (9.5-21.9) mm Hg (*P* < 0.0001), indicating reduced intraventricular pressures during filling (Table 2). Lower EDP postprocedure is consistent with the relief of outflow obstruction, which likely reduced intra-LV pressure throughout ejection and facilitated improved early diastolic relaxation. LV EDV decreased from 132 (112-150) to 120 (103-141) mL (*P* < 0.0001); however, this change was modest, and clinical filling status (assessed by EDP) actually improved. Importantly, diastolic capacitance, assessed by EDV at an EDP of 15 mm Hg, remained unchanged (120 [105-144] vs 124 [92-154] mL, *P* = 0.65) (Table 2). Thus, although TAVR reduced filling pressures, it did not alter intrinsic diastolic compliance within 24 hours.

## Discussion

This study provides clinical evidence that patients with severe AS exhibit a chronic activation of the Anrep effect, and that this phenomenon is rapidly reversible after TAVR. Key findings are summarized in the Central Illustration. Before intervention, our patients had hemodynamic characteristics, high LVESP, elevated contractility (steep ESPVR with high Ees), and prolonged ejection time that fit the profile of an Anrep effect maintaining cardiac output under conditions of excessive afterload. Following TAVR, we observed an immediate "unwinding" of these adaptations in all patients: LVESP and afterload fell, contractility indices diminished, and systole shortened substantially (Table 2). This triad of changes is the hemodynamic mirror image of the Anrep effect and confirms that the mechanism supporting stroke volume in AS was deactivated once the outflow obstruction was relieved.

**Figure 5:**
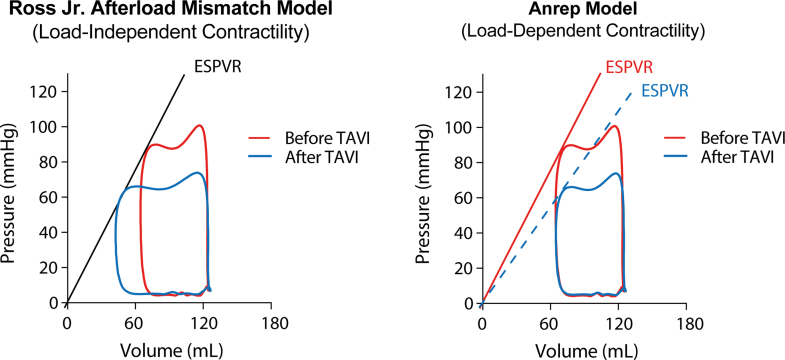
**Conceptual Models of Afterload-Contractility Coupling in Aortic Stenosis** Pressure-volume loops before (red) and after (blue) TAVR are shown under 2 theoretical frameworks. (Left) In the Ross Jr Afterload Mismatch Model, contractility is assumed to be load-independent, resulting in a fixed ESPVR slope. Unloading shifts the operating point along this unchanging slope, leading to increased SV and EF. (Right) In the Anrep model, contractility dynamically adapts to afterload. Here, the ESPVR slope is elevated under pressure overload and normalizes after TAVR. Despite a substantial reduction in contractility and systolic pressure, SV and EF remain similar. This highlights how energy-saving adaptations in load-dependent contractility can preserve output under high afterload and explains why SV and EF do not improve after TAVR despite favorable hemodynamic changes. ESPVR = end-systolic pressure-volume relationship; TAVR = transcatheter aortic valve replacement.

**Central Illustration:**
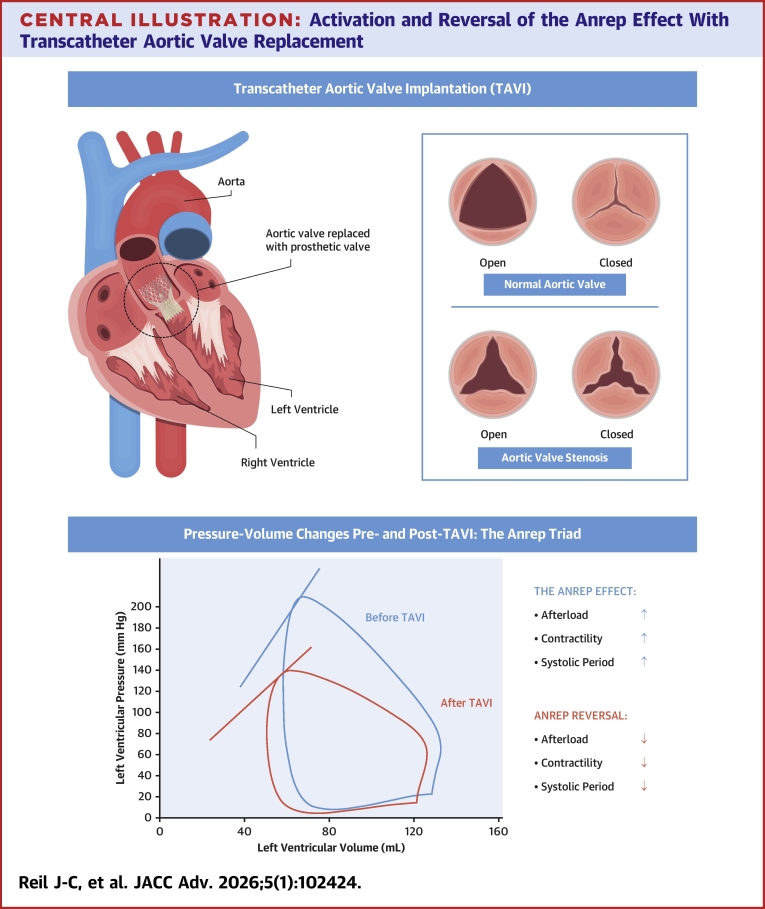
**Activation and Reversal of the Anrep Effect With Transcatheter Aortic Valve Replacement** This figure illustrates hemodynamic changes in aortic stenosis (AS) before and after transcatheter aortic valve replacement (TAVR). (Top) The left image shows the heart with a prosthetic valve in place after TAVR, whereas the inset on the right compares normal and stenotic aortic valve morphology. (Bottom) Left ventricular pressure-volume loops are shown before (blue) and after (red) TAVR. In AS, elevated afterload leads to activation of the Anrep effect, characterized by increased contractility and prolonged systolic duration. Following valve replacement, this compensatory state is reversed, with reduced afterload, contractility, and systolic period, thereby improving mechanical efficiency and lowering myocardial workload. Part of this figure is adapted from a licensed Adobe Stock image (#310015261, artist: Pepermpron), used under an Extended License with permission.

### Explaining the paradoxical EF response after TAVR

Our findings align with and help clarify prior observations in TAVR populations assessed noninvasively^22^ and invasively.^23^ Harrington et al^22^ reported that afterload indices significantly decreased following TAVR, yet measures of systolic function such as EF and mid-wall fractional shortening did not improve, and in some cases declined. This seemingly paradoxical result challenges the conventional expectation that afterload reduction should enhance systolic performance.^22^ This has sometimes been interpreted as contradicting the classic theory of afterload mismatch, proposed by John Ross Jr, which assumes that LV contractility (ESPVR, Ees) is independent of load.^24^ According to this model, excessive afterload (as in high-grade AS) restricts stroke volume and EF (“afterload mismatch”), and even enhanced preload (via the Frank-Starling mechanism) cannot compensate.^24^ Thus, unloading the ventricle through valve replacement should theoretically increase stroke volume and EF. However, our findings suggest that this assumption does not fully apply in patients with preserved EF and long-standing pressure overload.

The concept of a chronic Anrep effect offers an alternative physiological explanation. In this model, sustained afterload enhances contractility through afterload-dependent recruitment of myosin motor heads, increasing Ees and prolonging systolic ejection.^13^ This adaptation preserves stroke volume in the face of high resistance but does so at a significant energetic cost.^13^^,^^14^ These dynamics are particularly relevant when considering the nature of LV pressure generation in AS. Similar to oHCM, ejection in AS faces a fixed high resistance. To overcome this, the LV must generate elevated intraventricular pressure and maintain contraction for longer, effectively performing excess pressure work that contributes relatively little to forward flow. In other words, stroke volume is preserved not by efficiency, but by force, and at the expense of PE accumulation.

TAVR removes the mechanical stimulus sustaining this state. After intervention, contractility declines, ejection shortens, and workload drops, yet stroke volume and EF remain largely preserved. Rather than indicating a failure to improve function, this pattern reflects a transition from a hypercontractile, energy-consuming state to a normalized workload. Stroke volume is now maintained without excessive pressure generation. This load-dependent coupling between afterload and contractility is clearly illustrated in Figure 1: the ESPVR slopes before and after TAVR differ substantially, but EF is nearly identical. If contractility were truly load independent, the ESPVR would exhibit a fixed slope, leading to the expected rise in EF on afterload reduction, as originally proposed by Ross Jr^24^ This, however, is not what we observed (see schematic Figure 5). Rather than contradicting the afterload mismatch model, our findings refine it, supporting the notion that contractility is not fixed but dynamically modulated by mechanical afterload via the Anrep effect. The therapeutic benefit of TAVR in these patients lies not in an increase in EF, but in a substantial reduction in the energy required to maintain it.

### Normalization of systolic duration

The acute normalization of systolic timing is another important finding. Severe AS leads to a prolonged LV ejection period as the heart struggles to force blood through a narrowed orifice. We documented a ∼50 ms shortening of ejection time after TAVR (with heart rate correction) (Figures 2 and 3B), reflecting how quickly the systolic phase can normalize once the obstruction is relieved. This has practical implications: a shorter systolic duration at a given heart rate means more time is available for diastolic filling and coronary perfusion within each cardiac cycle. Thus, by eliminating the need for prolonged contraction, TAVR can indirectly benefit both diastolic function and myocardial perfusion, even if intrinsic diastolic compliance does not immediately improve. Prior studies corroborate these observations, showing that afterload reduction via TAVR improves coronary perfusion and diastolic function, consistent with the resolution of a sustained inotropic state.^25^, ^26^, ^27^ The observed drop in LVESP in our patients further supports this interpretation, as the heart was filling at lower pressures post-TAVR (Table 2), which may contribute to reduced pulmonary venous pressures and relief of heart failure symptoms.

### Energetic cost of the Anrep effect

Persistent activation of the Anrep effect in severe AS serves a compensatory purpose but comes at a substantial mechanical and energetic cost to the myocardium. Under normal physiological conditions, this inotropic reserve is transient and tightly regulated, supported by adequate energy production, optimal coronary flow reserve, sufficient oxygen supply, and efficient adenosine triphosphate (ATP) recycling systems. In AS, however, this balance is disrupted by shifts in substrate metabolism and mitochondrial dysfunction,^28^ reduced coronary flow reserve,^26^^,^^27^ and chronically elevated mechanical load (reflected by increased SW, PE, and PVA) (Table 2). These maladaptations drive up myocardial oxygen consumption and are associated with decreased high-energy phosphate availability, as evidenced by reduced phosphocreatine-to-ATP ratios.^25^^,^^26^^,^^29^^,^^30^ This creates a persistent mismatch between myocardial energy demand and supply. Notably, TAVR has been shown to facilitate recovery of phosphocreatine-to-ATP levels^25^^,^^26^^,^^29^ consistent with the reversal of the Anrep effect.

In our cohort, the observed decline in PVA after TAVR is particularly noteworthy. A lower PVA at comparable levels of external work implies an improvement in myocardial oxygen economy. This may lead to better oxygenation of the myocardium, particularly in patients with coexisting coronary artery disease, and could help reduce the risk of ischemia or demand-related dysfunction. Despite the favorable reduction in PVA, mechanical efficiency (SW/PVA) remained unchanged.

### Reversing the Anrep effect: impact of LV unloading

It is important to consider whether the observed decline in contractility after TAVR could be explained by mechanisms other than reversal of the Anrep effect. One possibility is that systemic catecholamines decrease postprocedure, perhaps due to relief of stress, thereby lowering inotropy. However, a reflex drop in adrenergic tone would typically be associated with prolonged systole, as reduced contractile force generally requires more time for ejection.^31^ In contrast, we observed a significant shortening of systolic duration post-TAVR, making this explanation less likely. Changes in heart rate were modest, and all rate-dependent parameters were corrected accordingly, ruling out a major contribution from the Bowditch effect (force-frequency relationship). In addition, neither the Frank-Starling mechanism nor the slow force response^32^^,^^33^ can account for the observed changes, as preload (EDV) does not influence contractility (ie, the slope of the ESPVR).^34^ The Gregg effect, which links coronary perfusion to contractility,^35^^,^^36^ is also unlikely to be a dominant factor in this context, as it typically has minimal impact in well-perfused, blood-perfused myocardial preparations.^37^

### Mechanistic basis and reversibility of the Anrep effect

The Anrep effect is a contractile adaptation triggered by the increased afterload, mediated in part by the recruitment of myosin motors from a dormant, energy-conserving reserve within cardiomyocytes.^13^ Under baseline conditions, a large proportion of myosin heads (∼55%) reside in this inactive conformation.^38^^,^^39^ When afterload rises, more of these motors transition into an active state, increasing actin-myosin interactions and augmenting force generation.^13^

Physiologically, this recruitment is typically brief, but in the setting of sustained pressure overload, as seen in oHCM,^13^ and plausibly in AS, this mechanism can become persistently (chronically) activated. Sustained wall stress maintains a greater fraction of myosin heads in the force-generating state, thereby increasing contractility but at a considerable energetic cost. As a result, myocardial oxygen demand rises, ventricular wall stress increases, and structural remodeling progresses, hallmarks of advanced AS.^28^ These features are consistent with chronic activation of the Anrep effect.^13^^,^^14^

TAVR disrupts this cycle by relieving the mechanical trigger for myosin activation. As afterload falls, the myocardium no longer needs to sustain elevated inotropy: myosin heads can return to their resting state, energy consumption declines, and systolic ejection shortens. The immediate changes we observed, including reduced contractility, briefer systole, and lower PVA (Table 2), are all consistent with a reversal of load-induced myosin recruitment.

This contractile plasticity helps explain how efficiently the ventricle adapts once afterload is removed.^25^^,^^26^^,^^29^ Over time, such unloading may facilitate structural regression as well. Longitudinal studies have demonstrated that after TAVR, LV hypertrophy regresses and diastolic function improves within months.^40^^,^^41^ Although our present analysis focuses on acute changes, the reversibility of myosin recruitment may represent an early mechanistic step in this broader remodeling process.

### Study Limitations

A primary limitation of this study is its short-term scope: we captured acute hemodynamic changes within 24 hours of TAVR but did not assess longer-term myocardial remodeling. Future longitudinal follow-up of this cohort (eg, 6-12 months) will determine whether the acute reversal of the Anrep response translates into sustained improvements in LV structure (eg, regression of hypertrophy or fibrosis) and function (eg, diastolic performance). In addition, our cohort consisted predominantly of patients with preserved EF; the interplay between afterload relief and intrinsic contractility may differ in patients with reduced EF or low-flow, low-gradient AS, where baseline myocardial reserve and adaptive mechanisms vary. Thus, our findings apply primarily to patients with classic high-gradient AS and normal systolic function.

Although this study did not include a contemporaneous healthy control group, previously published reference values from healthy individuals reported in Reil et al.,^11^ using the same echocardiography-based PV analysis, can offer context. In that reference cohort (median age: 49 years), Anrep-related indices were consistently lower than in our pre-TAVR patients: LVESP was 136 mm Hg, Ea 1.67 mm Hg/mL, Ees 2.5 mm Hg/mL, and SET 345 ms (SET_c_ 376 ms). Energetic parameters also differed: healthy individuals had a median SW of 7,062 mmHg mL, a PVA of 10,732 mmHg mL, and a mechanical efficiency of 67%. Following TAVR, most of these parameters shifted toward this healthy reference range, suggesting that the intervention helps normalize both hemodynamic and energetic stress. Still, this comparison is descriptive only, as no formal statistical matching was performed.

## Conclusions

In patients with severe AS, chronic pressure overload results in persistent activation of the Anrep effect, a contractile adaptation that enhances force generation and prolongs systolic ejection in response to elevated afterload. We demonstrate that TAVR acutely reverses this adaptive state. After intervention, patients exhibited reduced afterload (LVESP, Ea), lower contractility (decreased Ees, increased ESV_150_), and shorter systolic duration (SET), yet maintained stroke volume and EF. This immediate “backing off” of the ventricle from its high-pressure, high-contractility state supports the interpretation that unchanged EF after TAVR reflects a normalization of workload, not a lack of treatment effect, such that energy costs required for the same cardiac output are significantly lowered, thereby restoring energetic balance. These results identify the chronic Anrep effect as a clinically relevant, load-dependent mechanism in AS and highlight its reversibility post-TAVR.

Perspectives **COMPETENCY IN MEDICAL KNOWLEDGE:** The clinical takeaways from this study are 3-fold: First, patients with severe AS can maintain near-normal EF by activating an intrinsic afterload-dependent mechanism, the Anrep effect, which increases contractility and prolongs SET. This compensation, however, comes at a high energetic cost. Second, TAVR effectively unloads the ventricle and acutely deactivates this mechanism, as shown by immediate reductions in pressure, contractility, and SET, changes that are beneficial even if they do not translate into higher EF. Third, comprehensive PV loop analysis is valuable for capturing these subtleties. It helps explain why an intervention improves patient status (eg, reduced LV pressure and filling pressure) even when a standard measure like EF remains unchanged. For interventional cardiologists and imaging specialists, incorporating assessments of SET/SET_c_ as a timing biomarker (eg, Doppler-derived ejection time) and Ea/Ees (single-beat elastance calculations) as coupling/contractility markers could enrich post-TAVR evaluations.

**TRANSLATIONAL OUTLOOK:** In practice, an acute shortening of SET/SET_c_ and a drop in the Ea/Ees ratio may serve as early indicators of favorable unloading, with potential to forecast subsequent symptomatic improvement and structural regression observed on follow-up imaging. These measures complement EF and may help target longitudinal monitoring of remodeling and outcomes.

## Funding support and author disclosures

Dr Sequeira is supported by a research fund from Bristol Myers Squibb (unrelated to this work) and the Deutsche Forschungsgemeinschaft (DFG; project nos. 530849567 and 554784412). Dr Scholtz has received speaker honoraria from Bristol Myers Squibb unrelated to this work. All other authors have reported that they have no relationships relevant to the contents of this paper to disclose.
