# Supplementary material for: Activation of the Anrep Effect in Aortic Stenosis Pre-TAVR and Post-TAVR: An Echocardiographic Pressure-Volume Analysis
Source: JACC Adv. 2025 Dec 17;5(1):102424. doi: 10.1016/j.jacadv.2025.102424 (PMC12771300; doi:10.1016/j.jacadv.2025.102424)
Supplement: Supplemental_Material [file mmc1.docx]

**Supplemental Figures**

**
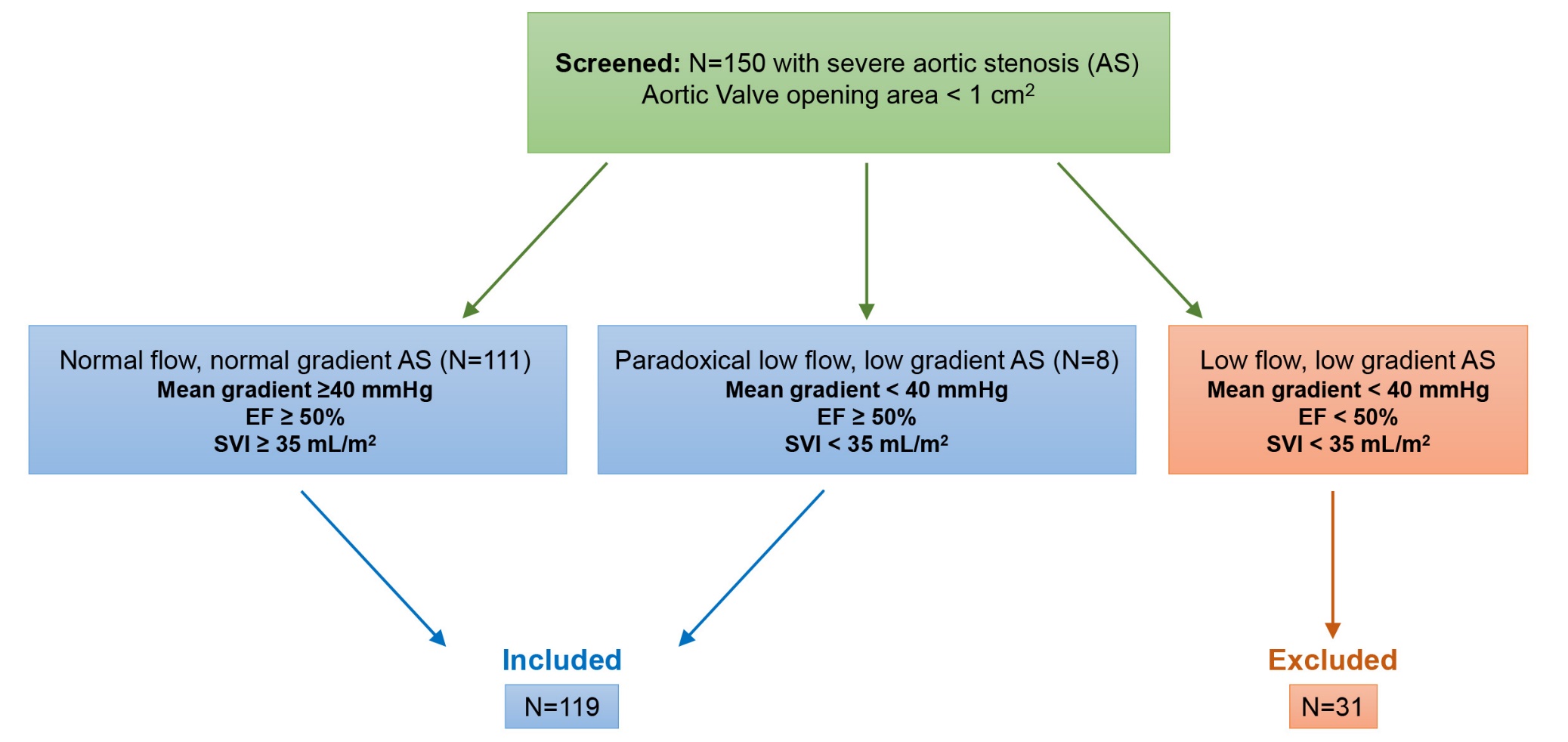
**

**Supplemental Figure 1. Study flow and cohort selection.** Of N=150 patients with severe aortic stenosis (AS; aortic valve area <1.0 cm^2^) assessed for eligibility, classical low-flow, low-gradient (LFLG) AS with reduced ejection fraction (EF <50%) were excluded (N=31). The included cohort (N=119) comprised normal-flow, normal-gradient (NF/NG) AS (N=111; mean gradient ≥40 mmHg, EF ≥50%, stroke-volume index [SVI] ≥35 mL/m^2^) and paradoxical LFLG AS (N=8; mean gradient <40 mmHg, EF ≥50%, SVI <35 mL/m^2^). Paired pre-/post-procedure hemodynamic–echocardiographic measurements were available in N=119 for most parameters.

**
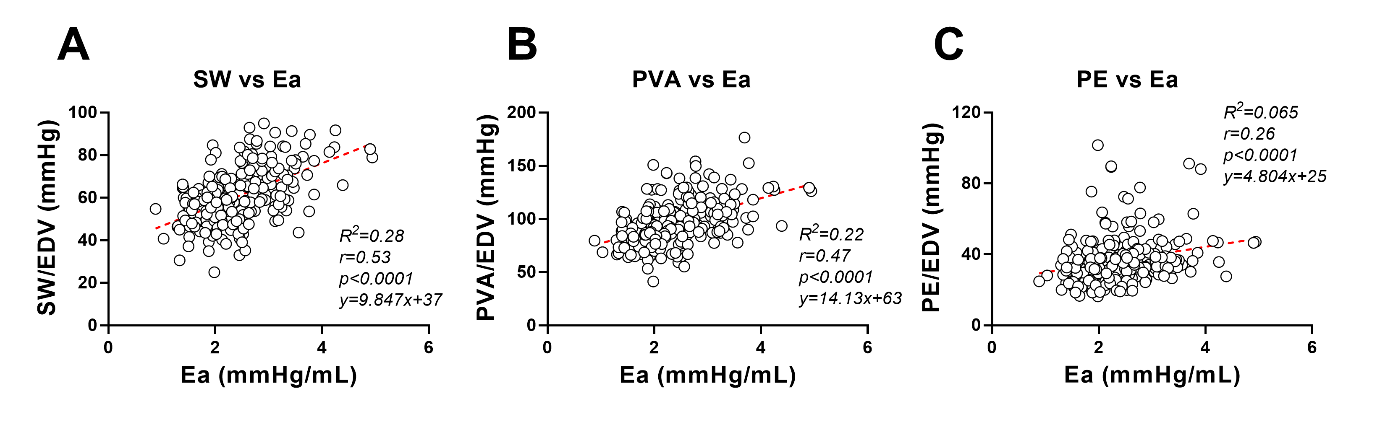
**

**Supplemental Figure 2. Correlations Between Afterload and Energetic Load Indices in Aortic Stenosis.** Scatter plots show linear regressions between effective arterial elastance (Ea, x-axis) and three energetic parameters (y-axis): stroke work per end-diastolic volume (SW/EDV), pressure–volume area per EDV (PVA/EDV), and potential energy per EDV (PE/EDV). Red dashed lines represent regression fits. These findings illustrate the link between afterload and myocardial energetic expenditure, further supporting the concept that the Anrep effect increases cardiac energy demand in AS.
